# Kinetics of CD19^+^ B-cell depletion post-rituximab in membranous nephropathy

**DOI:** 10.1093/ckj/sfaf153

**Published:** 2025-05-19

**Authors:** Mustafa Sevinc, Meshaal Shukkur, Patrick Hamilton, May Thet, Sebastian Bate, Omar Ragy, Durga A K Kanigicherla

**Affiliations:** Manchester Institute of Nephrology and Transplantation, Renal Department, Manchester, UK; Manchester University NHS Foundation Trust, Medicine Department, Manchester, UK; University of Manchester, Wellcome Centre for Cell-Matrix Research, Division of Cell Matrix Biology and Regenerative Medicine, School of Biological Sciences, Faculty of Biology, Medicine and Health Manchester, UK; Hull University Teaching Hospitals NHS Trust, Hull, UK; University of Manchester, Centre for Biostatistics, Division of Population Health, Health Services Research, and Primary Care Oxford Road Manchester, UK; Manchester University NHS Foundation Trust, Manchester Institute of Nephrology and Transplantation, Manchester, UK; Manchester University NHS Foundation Trust, Manchester Institute of Nephrology Transplantation, Manchester Royal Infirmary, Manchester, UK

To the Editor,

Rituximab (RTX) has shown efficacy in the treatment of primary membranous nephropathy (PMN) and is one of the recommended options in routine clinical practice [1]. However, remission rates following RTX treatment remain modest and optimal dosing is unclear, especially after the initial treatment. Although Kidney Disease: Improving Global Outcomes guidance suggests consideration of repeat dosing in some patients, it is unclear who may best benefit from this. Also, it is unknown if B cell depletion can be used to judge the efficacy of RTX therapy and how the durability of B cell depletion after RTX can influence remission and relapse. This study aims to assess the state of the peripheral B cell population after initial RTX treatment and association of B cell depletion with the immunological and clinical course in PMN.

Twenty-nine consecutive patients with biopsy-proven PMN were prospectively followed up after RTX treatment between 2019 and 2023. Two doses of rituximab, 1 g each, were administered on day 0 and day 14. A CD19 count of <5 cells/µ; is considered as depletion. A Euroimmun (Lübeck, Germany) enzyme-linked immunosorbent assay was used for measurement of anti-PLA2R. Detailed methods are described in the Supplementary Methods.

Baseline characteristics are presented in Table 1 and Supplementary Table S1. A total of 15 (52%) patients achieved partial remission with a median time to remission of 8.4 months (interquartile range 5.4–15.9). Relapses were observed in 6 (21%) patients during follow up. Table 1, Supplementary Table S1 and Supplementary Fig. S1 show baseline and follow-up parameters in the cohort.

**Table 1:** Baseline and follow-up parameters of the patients (*N* = 29).

	Outcomes				
Parameters	Baseline	3 months	6 months	9 months	12 months
Rescue treatment	NA	0	0	6	0
eGFR (ml/min/1.73 m^2^)	43 (31–57)	43 (33–63)	44 (32–65)	46 (38–62)	44 (36–59)
uPCR (mg/mmol)	893 (616–1185)	582 (320–840)	485 (238–791)	407 (126–645)	177 (66–447)
Albumin (g/dl)	23 (18–27)	26 (21–30)	27 (22–31)	30 (26–33)	31 (30–36)
CD19 (cells/µl)	155 (68–338)	2 (1–13)	13 (2–60)	22 (10–31)	69 (46–154)
Anti-PLA2Rab^a^ (RU/ml)	88 (34–154)	22 (12–39)	27 (11–47)	18 (4–39)	27 (5–30)
Remission, *n*/*N* (%)	NA	4/29 (14)	6/29 (21)	9/28 (33)	14/25 (56)

eGFR: estimated glomerular filtration rate; uPCR: urine protein:creatinine ratio; anti-PLA2Rab: anti-phospholipase A2 receptor antibodies.

Values are presented as median (interquartile range) unless stated otherwise.

a In 26 patients with anti-PLA2R-positive PMN.

CD19 depletion was achieved in all patients at 1 week and at 2–3 weeks. CD19 reconstitution was observed in 6% at 4–6 weeks, 25% at 2–3 months and 60% at 4–6 months from the first RTX dose. Anti-PLA2R levels decreased through 6 months from baseline but remained at a similar level until 12 months. Supplementary Fig. S2 depicts changes in anti-PLA2R and CD19 at a given time point compared with the previous time point. There was a statistically significant positive correlation between changes in CD19 and anti-PLA2R levels (*R* = 0.89, *P* = .0076). Notably, CD19 repopulation preceded the re-emergence of anti-PLA2R. The rate of partial remission at 1 year, which was modest at 52% patients, was similar to other published studies [2–5].

The CD19 cell count after RTX infusion can be used to assess if a patient had an adequate initial dose. We observed that 2 × 1-g RTX doses consistently achieved this within days after infusion in all our patients. However, the durability of CD19 suppression is poor with reconstitution being noted in 60% of patients by 6 months. And this preceded the changes in anti-PLA2R antibodies.

Previous studies investigating this aspect of B cell population in PMN are summarized in Supplementary Table S2 [1–5]. B cell reconstitution occurs after initial depletion and its timing differs according to the dose of rituximab, primary disease, age and other factors [3]. Results from our study are comparable with these previous studies. In addition, our study provides detailed phenotypes at much shorter time intervals, changes with CD19 over time and the additional association of these with changes in anti-PLA2R. Cumulatively, we believe that these observations and associations can well explain the plateauing of serum anti-PLA2R levels beyond 3–6 months and suboptimal remission rates with standard 2 × 1 g RTX dosing in PMN.

These observations thus suggest that CD19 monitoring is a good predictor of response after RTX treatment and could be used to identify patients who need additional dosing at 3–6 months. This can be especially useful for patients with a shorter CD19 reconstitution time and equally important for patients with non-PLA2R–associated PMN where no options are currently available for routine immunological monitoring. Such additional dosing guided by CD19 monitoring could help improve remission rates in PMN.

## Supplementary Material

sfaf153_Supplemental_File
