# Supplementary material for: Kinetics of CD19+ B-cell depletion post-rituximab in membranous nephropathy
Source: Clin Kidney J. 2025 May 19;18(6):sfaf153. doi: 10.1093/ckj/sfaf153 (PMC12138344; doi:10.1093/ckj/sfaf153)
Supplement: sfaf153_Supplemental_File [file sfaf153_supplemental_file.docx]

**Supplementary Materials**

Insufficient CD19 depletion with standard rituximab dosing: A potential contributor to suboptimal outcomes in membranous nephropathy

Mustafa Sevinc, Meshaal Shukkur, Patrick Hamilton, May Thet, Sebastian Bate, Omar Ragy, Durga AK Kanigicherla

**Table of contents**

Supplementary Methods

Supplementary References

Supplementary Table S1. Baseline characteristics of the patients

Supplementary Table S2. Summary of previous studies investigating B-cell population in primary membranous nephropathy

Supplementary Figure S1. Serial CD19, anti-PLA2R, proteinuria and albumin levels following Rituximab treatment.

Supplementary Figure S2. Proportional change in CD19 count and anti-PLA2R titre compared to the earlier time point

# **Supplementary Methods**

Twenty-nine consecutive patients with biopsy proven primary membranous nephropathy were prospectively followed up after rituximab treatment between 2019 and 2023. 2 doses of rituximab, 1 gram each, were administered on Day 0 and 14. Standard clinical markers, anti-PLA2R, CD19 cell count (cells/µL) were monitored at 1-week, 2-3-week, 4-6-week, 2-3-month, 4-6-month, 8-10-month, and 12-month timepoints after initial dose.

Data was censored at last follow-up visit or at rescue immunosuppression. Pearson correlation was used to investigate the relationship between CD19 repopulation and anti-PLA2R levels. Continuous variables presented as median and interquartile range, and categorical variables presented as number and percentage. All analysis was done in R Stats version 4.3.3.

# **Supplementary References**

1. Cravedi, P., et al., *Titrating rituximab to circulating B cells to optimize lymphocytolytic therapy in idiopathic membranous nephropathy.* Clin J Am Soc Nephrol, 2007. **2**(5): p. 932-7.

2. Ramachandran, R., et al., *CD19 Targeted Low-Dose Rituximab Is Effective in the Management of Refractory Phospholipase A2 Receptor Antibody-Associated Membranous Nephropathy.* Kidney Int Rep, 2017. **2**(1): p. 89-90.

3. George, J., et al., *Clinical Response and Pattern of B cell Suppression with Single Low Dose Rituximab in Nephrology.* Kidney360, 2020. **1**(5): p. 359-367.

4. KARGUPTA, A., Mukhopadhyay, P , Kataruka, M , Dasgupta, S , Rudra, A , Mukherji, A , Biswas, AK , Khan, MI, *COMPARATIVE STUDY BETWEEN SINGLE DOSE RITUXIMAB THERAPY VERSUS MULTIPLE DOSE REGIMEN IN IDIOPATHIC MEMBRANOUS NEPHROPATHY AND ITS CORRELATION WITH PERIPHERAL BLOOD CD19 COUNT*, in *World Congess of Nephrology*. 2023, KI Reports. p. S1-S473.

5. Teisseyre, M., et al., *Rituximab Immunomonitoring Predicts Remission in Membranous Nephropathy.* Frontiers in Immunology, 2021.

**Supplementary Table S1.** Baseline characteristics of the patients

|  | Baseline |  |
| --- | --- | --- |
| Median age (IQR), years | 56 (37-73) |  |
| Sex, female, % | 34 |  |
| Indication for rituximab, n(%)  First line  Second line  Relapse | 7 (24)  7 (24)  15 (52) |  |
| AntiPLA2R positivity, % | 90 |  |
| Median follow-up, months (IQR) | 13.5 (8.4-23.9) |  |

**Supplementary Table S2**. Summary of previous studies investigating B-cell population in primary membranous nephropathy

| **Study** | **n** | **1^st^ line vs 2^nd^ line** | **Follow-up** | **Rituximab dose** | **Remission** | **CD19** | | |
| --- | --- | --- | --- | --- | --- | --- | --- | --- |
|  |  |  |  |  |  | **1^st^ month** | **3^rd^ month** | **6^th^ month** |
| Remuzzi et al, 2002 | 100 | 1^st^ line 68%  2^nd^ line 32% | 29 months*** | 375 mg/m^2^ x4 until Oct 2005, then with CD19 monitoring | Complete 27%  Partial 38%  No remission 35% | NA | NA | NA |
| Fervenza et al, 2008 | 15* | 2^nd^ line 50% | 12 months | 1 gm two weeks apart | Complete 21.4%  Partial 42.8% | 5.6±6.7cells | 36±46 cells | 110±97 cells |
| Fervenza et al, 2010 | 20** | 1^st^ line 100% | 24 months | 375 mg/m^2^x 4 | Complete 22.2%  Partial 66.6% | 0.0±0.0 cells | NA | 35.8±64.2 cells |
| Mathew et al, 2023 | 4 | NA | ?1 year | Low dose | Complete 50 %  Partial 25% | NA | NA | NA |
| Ramachandran et al, 2017 | 6 | 2^nd^ line 100% | Min 6 months | 100 mg | Complete 50% at 6^th^ month | <1% after 1^st^ infusion, timing is not clear. The mean time for CD19 reconstitution was 2.17 ±1.17 (median 2, range 1–4) months. Reconstitution has not been defined | | |
| George et al, 2020 | 5 | 1^st^ line 100% | 12 months | 100 mg | Complete 40%  Partial 20% | 5±3.7 cells | 48.4±8.9 cells | 79±12.3 cells |
| Kargupta et al, 2023 | No detail | No detail | 6 months | 375 mg/m^2^ single dose in single dose group, two doses 7 days apart in multiple dosage group | CR or PR  58.3% in single dose, 61.5% in multiple dose group | 0.04% | 2% | 11.43% |
| Teisseyre et al, 2021 | 68 | No detail | 12 months | 1 gm two weeks apart | CR or PR  60% at 12 months | NA | 2 (0.0-7.0) | 22.5 (3.0-52.2) |
| Guo et al, 2022 | 35 | 2^nd^ line 91.5% | 6 months | Mostly, 375 mg/m^2^ x4  or 1 gm two weeks apart | CR or PR  20% | NA | 1.0 (0.0-3.0)/mm | 25.50 (3.75-57.75)/mm |
| Seitz-Polski, 2019 | 28 (NICE), 27 (GEMRITUX) | GEMRITUX first line | 6 months | NICE: 1 gm two weeks apart, GEMTIRUX: 375 mg/m^2^ at 1 week interval | CR or PR  NICE 64%, GEMTIRUX 30% | NA | NICE 0.0 (0.0-2.0)/mm^3^ GEMTIRUX 16.5 (2.5-31) mm^3^ | NICE 5.0 (1.8-48.5)/mm^3^, GEMTIRUX 63.0 (37.0-115)/mm^3^ |

*14 completed follow up, ** 18 completed follow up, *** median follow-up time after rituximab
